# Enhancing the Understanding of Paternal Involvement in Childcare and Its Impact on Maternal Disciplinary Practices

**DOI:** 10.2188/jea.JE20240235

**Published:** 2025-05-05

**Authors:** Hong Pan

**Affiliations:** Department of Health Management, Qingdao Hengxing University of Science and Technology, Qingdao, China

To the Editor:

The article “Paternal Involvement in Childcare and Housework and Mothers’ Spanking: A Longitudinal Study of Newborns in 21st Century Japan” in *Journal of Epidemiology* provides an insightful analysis using data from the 21st Century Japanese Newborns Longitudinal Survey, employing multivariable adjustments and stratified analysis.^1^ As social educators, we are encouraged to see the authors effectively demonstrate the potential impact of active paternal involvement in childcare and housework on reducing maternal spanking. The research highlights the role of fatherhood in family ethics resonating deeply with readers. This letter aims to acknowledge the contributions of this research while discussing areas for careful interpretation and further exploration.

First, the study reveals important associations between paternal involvement in childcare and housework and maternal spanking through statistical data and logistic regression analysis. However, while statistical models can reveal relationships between variables, they cannot fully capture the complexities of real-life situations. For instance, family dynamics, cultural background, and social support can significantly vary among different families.^2^ It is necessary to increase sample diversity to cover a broader population in future research.^3^

Second, the reliance on maternal reports led to a final analysis sample that included fewer than half of the original participants, highlighting the need to address participation barriers.^3^ Understanding the reasons for these barriers is crucial for developing strategies to improve future research participation and compliance. If many participants withdraw due to time conflicts or excessive burden, simplifying questionnaires or conducting phased surveys could reduce the time and effort required from participants.^4^ Establishing regular communication mechanisms, providing support and guidance, and addressing participants’ questions can also enhance their willingness to participate. Additionally, offering flexible survey schedules to allow participants to choose convenient times to complete the survey is important.

Third, despite the consideration of common demographic subgroup differences, additional factors, particularly community-provided social support networks, such as neighbor relationships, friends, and family support, should be further explored. These factors can alleviate maternal stress and consequently reduce spanking. Community childcare resources, such as daycare centers and childcare groups,^5^ can reduce the occurrence of spanking. Conducting sensitivity analyses to evaluate the impact of unmeasured confounding factors on the results ensures the reliability of research conclusions and enriches our knowledge and assists in tailoring future strategies to encourage paternal involvement in childcare and housework, thereby reducing maternal stress and spanking.

This study also emphasizes the design and testing of interventions to encourage more active paternal involvement in childcare and housework to reduce maternal stress and spanking. Incorporating professional social work practices—especially in rural areas, where social workers can form or connect community groups to strengthen network support, provide emotional support, and share experiences—can guide mothers to mental health services, such as stress management programs and counseling. Advocating for an integrated approach that addresses both social and psychological health in intervention research can change the current situation.

We are grateful that the authors emphasize the importance of the role of fatherhood in family ethics and the influence of the social environment on family ethics. We look forward to the authors promoting their research results across broader cultural contexts, verifying and expanding the findings of this study, enhancing research credibility, and delving into areas such as emotional and mental health, cross-cultural comparisons, long-term follow-up, and specific mechanism analyses. This will contribute significantly to family sociology research.
